# Naked Gold Nanoparticles and hot Electrons in Water

**DOI:** 10.1038/s41598-018-25711-2

**Published:** 2018-05-08

**Authors:** Khashayar Ghandi, Furong Wang, Cody Landry, Mehran Mostafavi

**Affiliations:** 1 Laboratoire de Chimie Physique, CNRS, Univ. Paris-Sud, Université Paris-Saclay, 91405 Orsay, France; 2 Department of Chemistry and Biochemistry, Mount Allison University, E4L1E4 NB, Canada

## Abstract

The ionizing radiation in aqueous solutions of gold nanoparticles, stabilized by electrostatic non-covalent intermolecular forces and steric interactions, with antimicrobial compounds, are investigated with picosecond pulse radiolysis techniques. Upon pulse radiolysis of an aqueous solution containing very low concentrations of gold nanoparticles with naked surfaces available in water (not obstructed by chemical bonds), a change to Cerenkov spectrum over a large range of wavelengths are observed and pre-solvated electrons are captured by gold nanoparticles exclusively (not by ionic liquid surfactants used to stabilize the nanoparticles). The solvated electrons are also found to decay rapidly compared with the decay kinetics in water. These very fast reactions with electrons in water could provide an enhanced oxidizing zone around gold nanoparticles and this could be the reason for radio sensitizing behavior of gold nanoparticles in radiation therapy.

## Introduction

There is a growing interest in the use of gold nanoparticles for a wide range of industrial, medicinal, and health applications^1–3^. Developing technologies and applications based on gold nanoparticles requires fundamental understanding of how they affect their environment.

Combination of the under- coordinated surface atoms and large surface-area-to-volume ratio is the reason for large numbers of surface atoms that play a significant role in the unique properties of nanoparticles^4,5^. Availability of such surface atoms is an important factor for many applications that rely on the reactivity of nanoparticles and their interaction with light^6–8^. Gold nanoparticles in which all surface atoms are available (naked gold nanoparticles), in particular when soluble in water, have been proposed for novel applications in radiation therapy and energy production^6–8^, based on their effects on their proposed luminescence under Cerenkov radiation^8^, and indirect observation of the fastest electron capture rate constant in water for pre-solvated electrons^6,7^. Despite the consistent picture of ultrafast electron capture via indirect scavenging measurements and computational results^6^, there has never been any direct evidence of such efficient pre-solvated electron capture by gold nanoparticles in water. Also there has never been any report of the effects of gold nanoparticles on Cerenkov spectrum in solutions by picosecond electron pulses. This paper provides direct evidences for both of these aspects for the first time.

## Results and Discussion

Picosecond pulse radiolysis is a powerful tool for investigating the dynamics of pre-solvated electron in liquids including water^9,10^. In the present work, this technique was extended to naked gold nanoparticles stabilized with ionic liquids in water. The ionic liquids in water provide novel environmentally friendly synthetic methods for making long-term stable (more than six months) metal nanoparticles in solvents without using covalent bonds nor with strong intermolecular interactions with the gold atoms on the surface^6,7^. The radiolysis from ionizing radiation, typically used in radiation therapy, in water leads to production of many transient intermediates and stable products^11^.

However, the initial stage of radiolysis involves ionization of water molecules to generate free electrons and H_2_O·^+^ ^12^. There are very few species that can react at very low concentrations with electrons before they solvate in water.

To test the proposed fast capture of pre-solvated electrons by gold nanoparticles and to measure the effect of gold nanoparticles at very low concentration on Cerenkov spectrum in water, the absorption of light over a wide range of wavelengths was observed as a function of time and wavelength immediately after electrons were passed through the sample at different time delays. It should be noted that such experiments are extremely difficult for the following reasons: (1) We need to compete with electron solvation in water which is complete in a ps. That means the experiments should be done at lower temperatures. The solvation of electron becomes faster at higher temperatures. Therefore, the ideal temperatures are the temperatures closer to freezing point. (2) Our previous experiments show that the naked nanoparticles in water stabilized by ionic liquids^6,7^ are stable for more than six months but only at temperatures higher than 10 °C. This means we are dealing with a delicate balance of intermolecular interactions among water molecules. As such, we need to choose the right temperature window, which is very limited. For these experiments we did not have access to lower temperatures than 23 °C. (3) Correct concentration ranges of gold nanoparticles should be selected because the gold nanoparticle solutions absorb very strongly, and this could interfere with the observation of hydrated electron spectrum. The 2 × 10^−7^ M is the largest concentration we could use because above this concentration the solution absorb so much light that no probe light could in practice pass through the solution. In principle, there is no lower concentration limit but the lower the concentration; the weaker the scavenging of electrons and when the concentration is too low, the solution will behave as water. (4) It is necessary to explore a wide range of wavelengths for these investigations depending on the concentration. We need to choose a wavelength for kinetics studies that is far from gold nanoparticle absorption and the high intensity part of the Cerenkov emission spectrum. (5) We need to carefully measure the Cerenkov spectrum and compare the Cerenkov spectrum from low concentration solutions of gold nanoparticles with the Cerenkov spectrum from water and other types of solutions because this can allow us to use the changes in the Cerenkov spectrum as a potential analytical tool.

**Figure 1:**
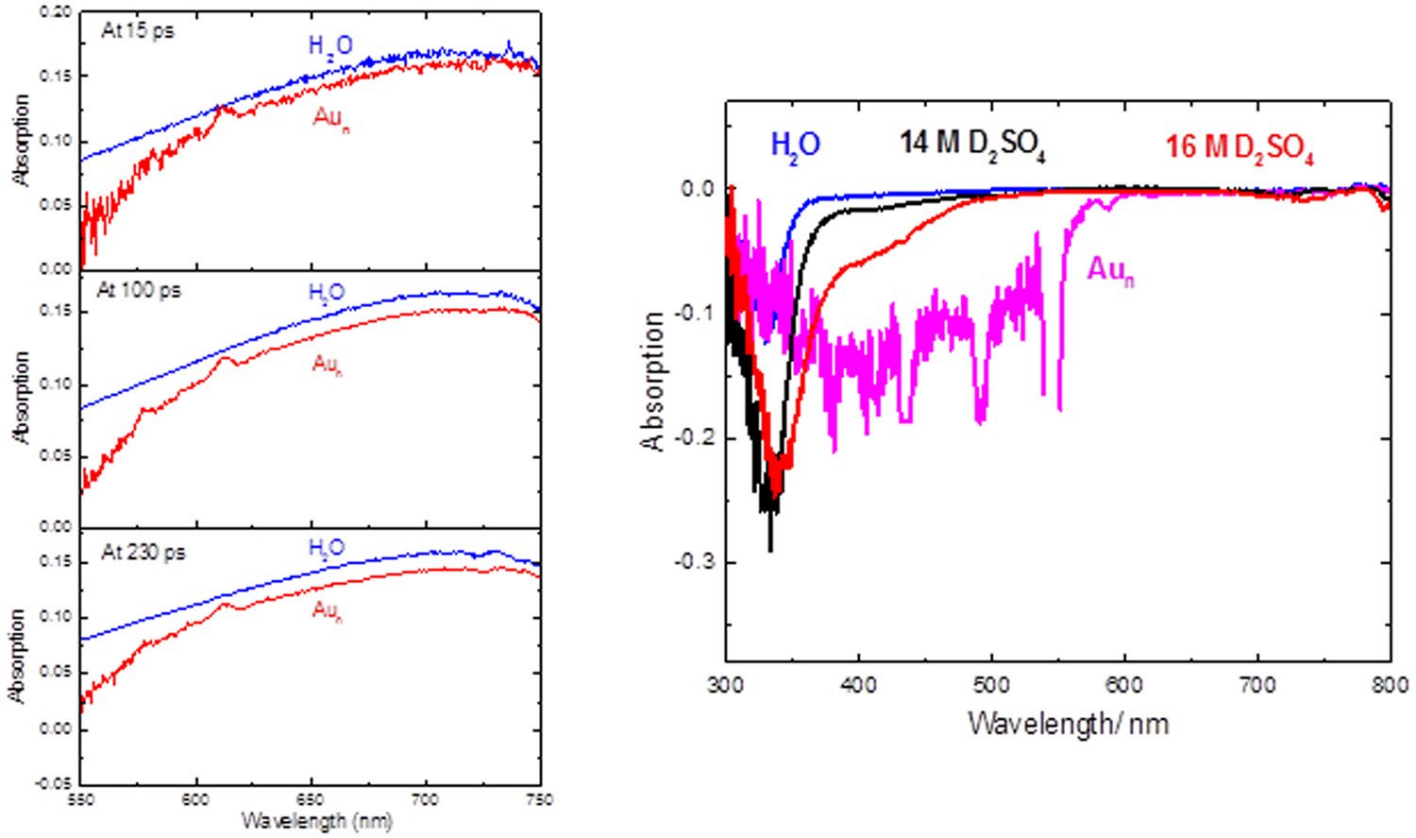
(**a**) (left). Transient absorption spectra of hydrated electron recorded 15 ps, 100 ps and 230 ps after the passage of the 7 ps electron pulse for water and solutions of 3.3 × 10^−8^ M naked 15 nm gold nanoparticles in water (Au_n_) at 23 °C. The transient spectra at different times are scaled for clarity. Details of gold nanoparticle solutions are described in the methods section. (**b**) (right). Cerenkov radiation absorption spectrum for different solutions are compared. Note that the gold nanoparticle solution has more than eight orders of magnitudes smaller concentrations than the D_2_SO_4_ solutions.

The primary observed species was e_(aq)_^−^ as can be seen in Fig. 1a ^13,14^. While the shape of the hydrated electron spectrum is not changed, the shape of the Cerenkov spectrum is significantly changed even at very low concentrations of gold nanoparticles (Fig. 1b). We compared the Cerenkov spectra of a few solutions in Fig. 1b. We used water as the reference. Other than water and gold nanoparticle solutions that we sought to compare, we wanted to use a very high concentration of an ionic solute with minimal light absorption in the near UV and visible range. We used both D_2_SO_4_ and H_2_SO_4_ (where we had the highest ionic concentration available) but did not observe significant difference between the two. The D_2_SO_4_ data was with a higher statistics and is shown in Fig. 1b. The larger uncertainty of Cerenkov spectra for gold nanoparticle solutions is due to strong light absorption of the solution.

The Cerenkov radiation is the light emitted when a charged particle (in this case an electron) passes through an electrically polarizable medium at a greater phase velocity than that of light in that medium. Figure 1b shows that Cerenkov spectrum is a sensitive function of the solutions’ concentrations and chemical structure. This has never been reported for liquid solutions. A notable observation is the very large effect of a very small concentration of gold nanoparticles on Cerenkov spectrum. It is clear that the Cerenkov spectra depends on chemical nature of the solution. Also maximum effects can be seen for solutions of heavy metal nanocrystals. Considering the recent interests in using the combination of Cerenkov radiation and nanotechnology for medical applications^8^, our results have significant implications for future medical application, in radiation therapy.

**Figure 2:**
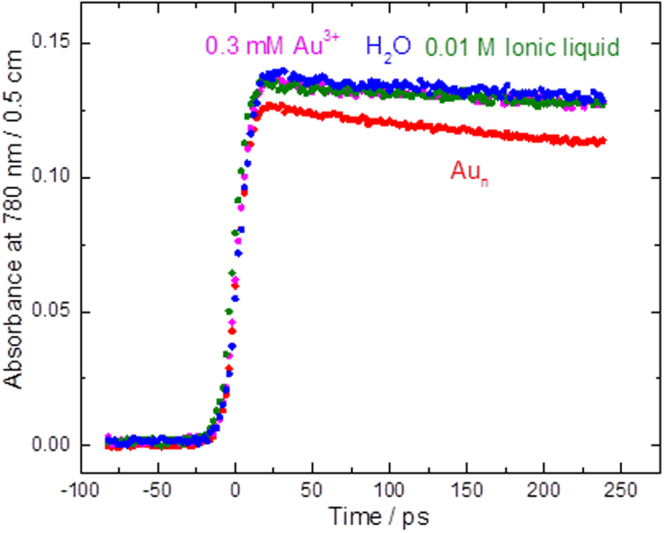
Recorded kinetics at 780 nm in water up to 250 ps for a solution of ionic liquid in water at the same concentration used to stabilize gold nanoparticles, solution of 3.3 × 10^−8^ M naked 15 nm gold nanoparticles in water (Au_n_), and solution of 0.3 mM Au^3+^ in water (Au^3+^ was used as precursor of gold nanoparticles) at 23 °C.

Figure 2 shows the time dependence of absorbance immediately after 7 ps electron pulse. The initial transient increase in absorbance at 780 nm (Fig. 2) is due to formation of hydrated electron (Fig. 1). The 780 nm is selected because: 1) it is far from the Cherenkov radiation spectrum and 2) it is far from the maximum absorption wavelength of gold nanoparticles.

As the ionic liquid stabilizers and gold ions don’t affect the initial yield of the hydrated electron significantly just after 7 ps electron pulses, it is clear that only gold nanoparticles at low concentrations caused a decrease in hydrated electron yield. This shows the pre-solvated electron is captured and it is the first direct evidence of the capture of pre-solvated electrons by gold nanoparticles.

Because of Cerenkov radiation under 600 nm and the strong absorption of gold nanoparticles solutions and hydrated electrons above this wavelength, it is almost impossible to detect any transient species using visible light as a probe (other than the hydrated electron)^15–20^.

**Figure 3:**
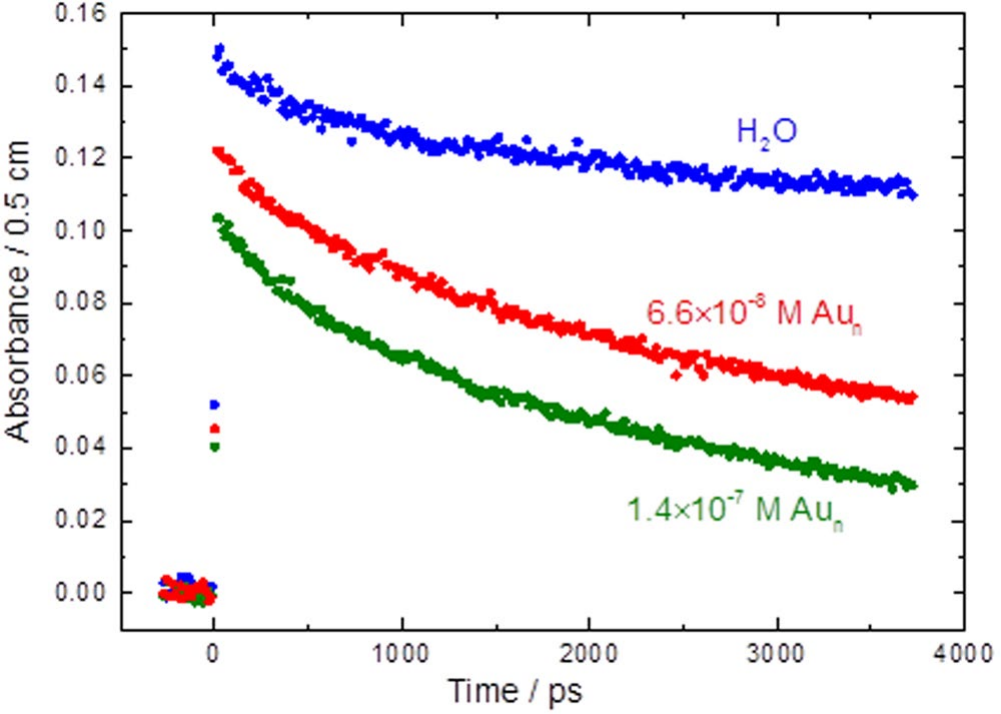
Recorded kinetics at 800 nm in water up to 4 ns for solution of 6.6 × 10^−8^ M (blue circles) and 1.4 × 10^−7^ M naked 15 nm gold nanoparticles in water (Au_n_) at 23 °C.

The decay of hydrated electron was also observed in ps (Fig. 2) and ns time scales (Fig. 3). The reason for using the larger concentrations of gold nanoparticles and longer time scale of the study here was to be able to observe the reaction kinetics of hydrated electron which is clear only in ns time scale for these solutions (as opposed to ps time scale used for experiments in Fig. 1). Due to the larger concentrations used for these studies, the light absorption at lower wavelengths make the uncertainty larger, hence the need to use a larger wavelength. Indeed our studies were done to above 1000 nm wavelength and the same decay were observed as the one shown at 800 nm in Fig. 2.

The slow decay observed in Figs 2 and 3 in water and low concentration solutions of Au^3+^ and ionic liquid in water are due to spur reactions of hydrated electron. The difference in the decay rate of the hydrated electron in water and in solutions of gold nanoparticles is due to reactions of hydrated electrons with gold nanoparticles. For all the solutions, other than the solutions of gold nanoparticles, the solvated electron lasts over several thousand ps, as in neat water. For solutions of gold nanoparticles, the solvated electron is reacting with gold nanoparticles and lasts only over several hundred ps.

These findings are promising when considering the use of gold nanoparticles in the field of radiation chemistry, including radiation therapy. There are two problem inherent in current radiation therapy techniques. The first problem is the occurrence of a bystander effect when applying radiation as radiation does not discriminate between healthy and malignant cancer cells, as such, healthy tissue is also destroyed in the process. Intensity-modulated radiation therapy (IMRT) uses multiple beams of radiation that converge on cancer cells; this increases the radiation intensity at the convergent point and decreases the collateral damage to healthy tissue however it does not eliminate it^21^. This limits the intensity of ionizing irradiation that could be used and thus limits the ability to thoroughly eradicate cancerous tissue. The gold nanoparticles used here provide an elegant resolution to this and depending on their radiation chemistry in solution can either be implanted into healthy tissue or cancer tissue to minimize the bystander effect as outlined below. The second problem is the occurrence of opportunistic infections which arise due to the weakening of the immune system by depleting blood cells of various subsets of lymphocytes when exposed to radiation^22^. Our gold nanoparticles are stabilized by an ionic liquid, that is a known antimicrobial agent, further increasing their attractiveness in radiation therapy research through a two-pronged approach^23^.

**Figure 4:**
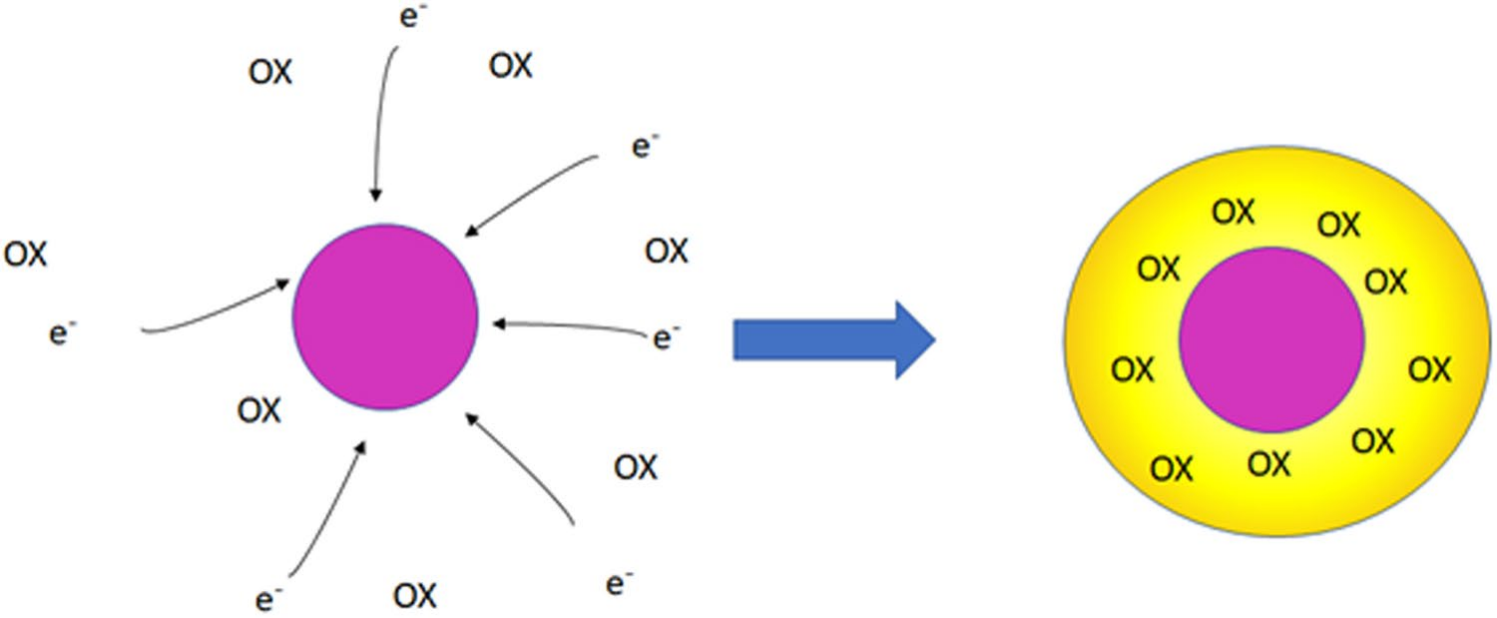
Schematic representation of enhanced oxidative damage by gold nanoparticles due to production of an oxidative zone around them.

As mentioned above, depending on the radiation chemistry of gold nanoparticles in solution, they could either be used in healthy or cancer tissues. If the nanoparticles only react with presolvated and solvated electrons and not by H_2_O·^+^ and OH· then the degree of recombination of electron and H_2_O·^+^ decreases significantly. This will then enhance the production of OH· radicals in close vicinity of gold nanoparticles (Fig. 4).

In this case, we will have an enhanced oxidative damage and therefore we can use gold nanoparticles in the cancer cells to enhance radiation effects locally within the cancer cells. This way a reduced ionizing radiation dose could have an enhanced effect and as such lower dose of radiation could be used.

In the second scenario, assuming that gold nanoparticles react with presolvated electrons and solvated electrons and the product of this reaction would then react with OH· radicals generated by H_2_O·^+^, a solution of gold nanoparticles could be injected into the tissue surrounding a tumor, which, as we see here, could act to capture pre-solvated electrons to reduce the burning of normal tissues and decrease the pain felt by the patient.

In both cases an understanding of mechanism of action of gold nanoparticles allow for proper placement of them that will then allow for more intense beams of radiation to be used on tumors, drastically decreasing the chance of recurrence by ensuring maximum destruction of malignant cells. This quenching of the pre-solvated electrons is essential as it has been found that their radiolytic yield can be far greater than that of hydrated electrons and can even attach to DNA and amino acids. It is crucial to capture the electrons at this stage^24,25^.

These results raise an important question: why did the gold nanoparticles at such low concentrations react so quickly with pre-solvated electrons? Our previous computational works suggests a couple factors that be playing a role in making the reactions extremely fast^6^: (1) Very large electron affinity and large negative free energy of electron capture of naked gold nanoparticles in water. The electron affinity of gold nanoparticles in water is the largest electron affinity ever reported. (2) Such a large affinity towards a free electron and the delocalized nature of the free electron means the reaction in water should be faster than diffusion limit. Therefore, the other factor is the large cross-section of nanoparticles, in particular when compared with small molecules.

The remarkable efficiency of naked gold nanoparticles for capture of pre-solvated electrons in water was demonstrated directly for the first time. The nanoparticles, even at extremely low concentrations, have a very significant effect on Cherenkov spectrum in water. The difficult conditions of the experiments did not allow us to observe the transient intermediates in the visible region after the capture of electrons, mostly due to interference with Cherenkov radiation. We invite theoretical chemists to investigate the electron capture rate computationally. In the future, we will develop techniques to enable us to observe transient intermediates from reactions with electrons and other free radicals with gold nanoparticles. The properties of the nanoparticles can be tuned by changing the particle size, i.e., by changing their cross sections in water and changing the surface-to-total atoms ratio^15^. As such, we are currently investigating the tunability with size of nanoparticles with respect to their reactivity with pre-solvated electrons, solvated electrons, and other transient intermediates. The implication of this work in radiation therapy is important because of its potential to provide a more effective radiation therapy with minimal side effects on healthy tissues.

## Methods

### Nanoparticle synthesis

Benzyldimethyltetradecylammonium chloride, bac-14 [TCI-America], is used as a protecting group and stabilizing agent. Sodium borohydride, NaBH_4_, is used as a reducing agent promoting the reduction of the gold [Au^III^ Au^0^] in water, as evidenced by the disappearance of the absorbance peak at 340 to 400 nm. The synthesis is carried out as follows: In a 250-mL round bottom flask, aqueous HAuCl_4_ [25 mL, 2.5 mmol] was stirred at 40 °C under a reduced atmosphere of nitrogen [Praxair 99.997%] for 15 minutes. Bac-14 [4 g, 10 mmol] was then added to 60 mL water and combined with the aqueous HAuCl_4_ solution to produce a yellow-solution. NaBH_4_ [800 mg, 21 mmol] was added to 15 mL of distilled deionized water and the resulting solution was added dropwise to the reaction mixture over several minutes. Reduction was instantaneous and the mixture was allowed to stir under mild heat for 2 to 3 hours. The solution was centrifuged and then the gold nanoparticles were dissolved in deionized water again (after washing). This way we made sure no gold ion remained in the solution, which was further confirmed by UV-Vis spectra.

### Characterization of nanoparticles

**Figure 5:**
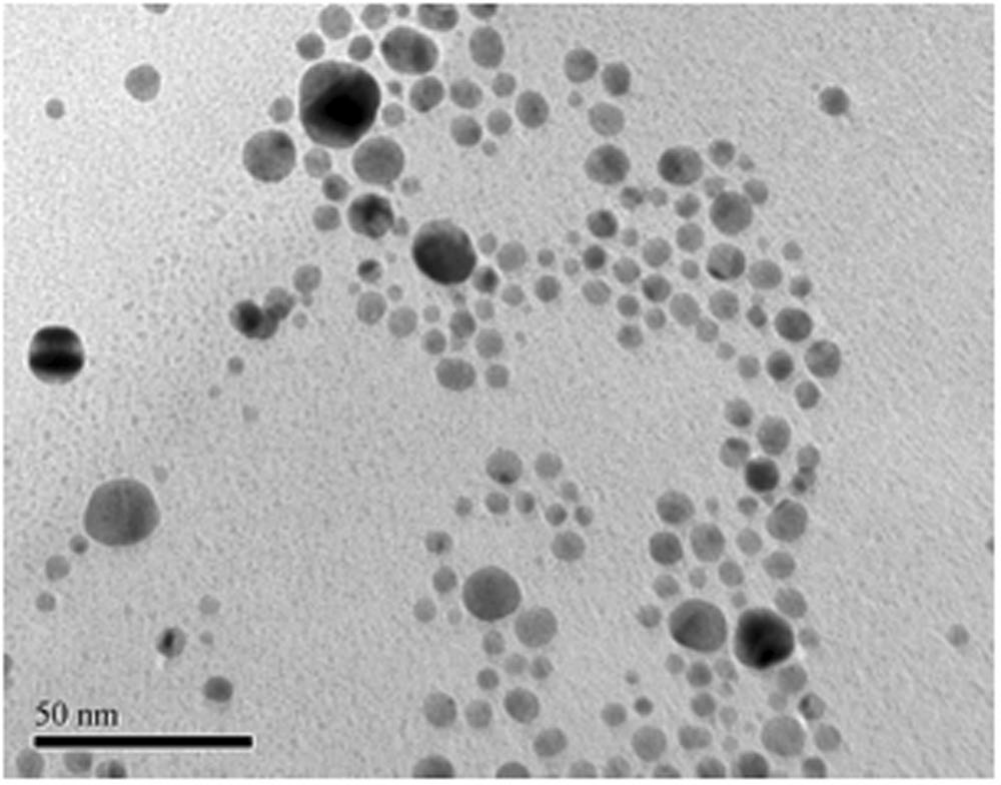
TEM image of as prepared gold nanoparticle. Average diameter is 15 nm.

Figure 5 is a TEM image of prepared gold nanoparticles.

### Pulse radiolysis

The formation and decay of the solvated electron and the observed transient species from reaction of pre-solvated electron and gold nanoparticles was followed using the pulse radiolysis system of the ELYSE accelerator (Paris-Sud University), which uses short pulses of electrons to produce and excite a fs broad supercontinuum to examine transient species with high time resolution. Two experimental areas developed at the ELYSE platform allow conducting pulse radiolysis studies on the sub-nanosecond to low nanosecond time scale, using the transient absorption pulse-probe set-up and nanosecond to the millisecond time scale, using the streak camera set-up. For this work only, the studies on the sub-nanosecond to low nanosecond time scale were performed. Laser (260 nm) driven Cs2Te photocathode allows production of short electron pulses with a typical half width of 7 ps and a charge of ~6 nC, and energy of ~7.8 MeV, at a repetition rate of 10 Hz. For the picosecond pump-probe experiments, the broadband supercontinuum (380–750 nm), generated by focusing a small part of the fundamental laser (780 nm) into a CaF2 crystal, and is split 60/40 probe/reference paths. Both probe and reference are coupled into optical fibers, transmitted to a spectrometer, and dispersed onto a cooled charged couple devicecamera. The sample cells used for the pump-probe measurements are 0.5 cm optical path length synthetic fused silica cells, through which the sample is continuously flown (ca. 20 cm^3^/min). The cells’ optical windows are of 200 μm thickness to minimize contributions to the signal from the transient species generated in quartz by the electron pulse. All kinetics presented in the work are calculated from averaging 10 transient maps with 15 electron pulses at each time delay step.

The dose deposited per pulse was determined from measurements of the absorbance of solvated electrons (${{\rm{A}}}_{{{e}^{-}}_{{\rm{aq}}}}({\rm{\lambda }},{\rm{t}})$) in water at 22.5 °C and verified before each series of experiments, considering the initial yield of the solvated electrons, measured at 10 ps, to be G(10 ps) = 4.5 × 10^−7^ mol/J, and its extinction coefficient ε_715nm_ = 19700 M^−1^·cm^−1^. All results presented herein were measured at an absorbed dose of close to 40 Gy per pulse. The samples were purged with argon gas continuously during the measurements.
